# Tuning the Microenvironment to Create Functionally Distinct Mesenchymal Stromal Cell Spheroids

**DOI:** 10.1007/s10439-023-03162-9

**Published:** 2023-02-21

**Authors:** Victoria L. Thai, Diego O. Candelas, J. Kent Leach

**Affiliations:** 1 Department of Biomedical Engineering, University of California, Davis, Davis, CA 95616 USA; 2 Department of Orthopaedic Surgery, UC Davis Health, 4860 Y Street, Suite 3800, Sacramento, CA 95817 USA

**Keywords:** Spheroids, Endothelial cell, Mesenchymal stromal cell, PEG-4MAL, Wound healing, Design of experiments

## Abstract

Mesenchymal stromal cells (MSCs) are under investigation for wound healing and tissue regeneration due to their potent secretome. Compared to monodisperse cells, MSC spheroids exhibit increased cell survival and enhanced secretion of endogenous factors such as vascular endothelial growth factor (VEGF) and prostaglandin E2 (PGE_2_), two key factors in wound repair. We previously upregulated the proangiogenic potential of homotypic MSC spheroids by manipulating microenvironmental culture conditions. However, this approach depends on the responsiveness of host endothelial cells (ECs)—a limitation when attempting to restore large tissue deficits and for patients with chronic wounds in which ECs are dysfunctional and unresponsive. To address this challenge, we used a Design of Experiments (DOE) approach to engineer functionally distinct MSC spheroids that maximize VEGF production (VEGF_MAX_) or PGE_2_ production (PGE_2,MAX_) while incorporating ECs that could serve as the basic building blocks for vessel formation. VEGF_MAX_ produced 22.7-fold more VEGF with enhanced endothelial cell migration compared to PGE_2,MAX_, while PGE_2,MAX_ produced 16.7-fold more PGE_2_ with accelerated keratinocyte migration compared to VEGF_MAX_. When encapsulated together in engineered protease-degradable hydrogels as a model of cell delivery, VEGF_MAX_ and PGE_2,MAX_ spheroids exhibited robust spreading into the biomaterial and enhanced metabolic activity. The distinct bioactivities of these MSC spheroids demonstrate the highly tunable nature of spheroids and provide a new approach to leverage the therapeutic potential of cell-based therapies.

**Supplementary Information:**

The online version contains supplementary material available at 10.1007/s10439-023-03162-9.

## Introduction

Mesenchymal stromal cells (MSCs) are commonly used for wound healing applications due to their differentiation capability and therapeutic secretome that reduces inflammation, enhances angiogenesis, and promotes tissue formation.^18,35^ Their versatile nature has been harnessed in numerous applications including myocardial infarction, bone regeneration, and cutaneous wound healing.^2,9,15^ Furthermore, the addition of endothelial cells (ECs) to MSCs increases angiogenic and osteogenic regeneration in critically sized bone defects.^6,28,29^ While monodispersed MSCs embody multifaceted roles in various therapeutic approaches, we and others have established the advantages of using MSCs as aggregates, also known as spheroids.^14^

Spheroids exhibit increased overall function due to improved cellular viability in harsh environments and enhanced secretion of endogenous growth factors (GFs).^12,16^ Among other factors, MSC spheroids secrete more vascular endothelial growth factor (VEGF) and prostaglandin E2 (PGE_2_), two important bioactive factors in wound healing.^37^ VEGF stimulates angiogenesis, while PGE_2_ mediates local inflammation and promotes the recruitment of cells that are critical for re-epithelialization.^23,30^ We previously reported a combination of microenvironmental conditions to form MSC spheroids with enhanced secretion of both VEGF and PGE_2_ for cutaneous wound healing.^25^ Spheroid size, oxygen tension, and inflammatory cues influence the MSC spheroid secretome. However, because our previous spheroid formulation was designed to simultaneously upregulate VEGF and PGE_2_ secretion, it does not fully capitalize on the therapeutic capabilities of MSCs. By developing individual spheroids, we can more precisely tune the conditions to maximize the secretion of a preferred growth factor for therapeutic use. These spheroids could be used in a modular approach to achieve the desired cytokine concentration. Furthermore, our previous approach is limited by its dependence on the responsiveness of host ECs for neovascularization. Individuals susceptible to chronic, nonhealing wounds are often diagnosed with diabetes, cardiovascular diseases, renal disease, or are obese. These debilitating diseases give rise to EC dysfunction, which causes native ECs to become unresponsive to potent bioactive factors secreted by spheroids and consequently hinders angiogenesis.^10^ The challenges presented by chronic wounds motivate the need for a therapy that promotes vascularization and epithelialization without depending on host ECs.

When co-cultured with endothelial colony forming cells (ECFCs), MSCs promote neovascularization, yielding stable vascular structures.^17,20,33,36^ However, this approach fails to fully address the critical need for epithelialization due to rapid cell death when transplanted in harsh microenvironments. To address this challenge, we engineered MSC spheroids with distinct secretomes and incorporated ECFCs to provide the basic building blocks for neovascularization. We hypothesized that MSC spheroids engineered with different therapeutic potentials (*i.e.*, maximizing VEGF or PGE_2_ secretion) containing ECFCs would promote vascularization and epithelialization. Herein, we used a Design of Experiments (DOE) multivariable analysis to engineer these unique spheroids and determine the interaction between multiple input variables shown to influence the MSC secretome. Following optimization, the functionally distinct spheroids were then entrapped in MMP-degradable poly(ethylene glycol) (PEG) hydrogels to explore spheroid interactions with their microenvironment. This study offers the unique opportunity to independently tune MSC spheroids and leverage them in a modular fashion to maximize specific growth factor secretion for regenerative therapies.

## Materials and Methods

### Cell Culture

Human endothelial colony forming cells (ECFCs) were isolated from human cord blood obtained through the UC Davis Cord Blood Collection Program (UCBCP).^24^ Cells were expanded in EGM-2 supplemented media (PromoCell, Heidelberg, Germany) with gentamicin (50 µg/mL; ThermoFisher, Waltham, MA) and amphotericin B (50 ng/mL; ThermoFisher) under standard culture conditions (37 °C, 5% CO_2_, 21% O_2_) until use at passages 7–8. Media changes were performed every 2 days. Human bone marrow-derived MSCs from two male donors (21-year-old, RoosterBio, Inc., Frederick, MD and 22-year-old, Texas A&M University, College Station, TX) were expanded without further characterization in standard culture conditions in minimum essential alpha medium (α-MEM; Invitrogen, Carlsbad, CA) supplemented with 10% fetal bovine serum (FBS; Atlanta Biologicals, Flowery Branch, GA) and 1% penicillin/streptomycin (P/S; Gemini Bio-Products, Sacramento, CA) until use at passages 4–5. Media changes were performed every 2–3 days. Diabetic human dermal microvascular cells (HMVECs) (Lonza, Walkersville, MD) from a single donor (66-year-old female) were expanded without further characterization in standard culture conditions in EGM-MV2 supplemented media (PromoCell) with gentamicin (50 µg/mL) and amphotericin B (50 ng/mL). Media changes were performed every 2 days. HaCaT cells (AddexBio Technologies, San Diego, CA), an immortalized human keratinocyte line from a single donor (62-year-old male), were expanded without further characterization in standard culture conditions in Dulbecco’s Modified Eagle Medium (DMEM; Invitrogen) supplemented with 10% FBS and 1% P/S. HaCaTs were used at passages 15–18. Media changes were performed every 2–3 days. Aliquots were derived from the same batch of serum to ensure consistency.

### Design of Experiments (DOE) Model

**Figure 1:**
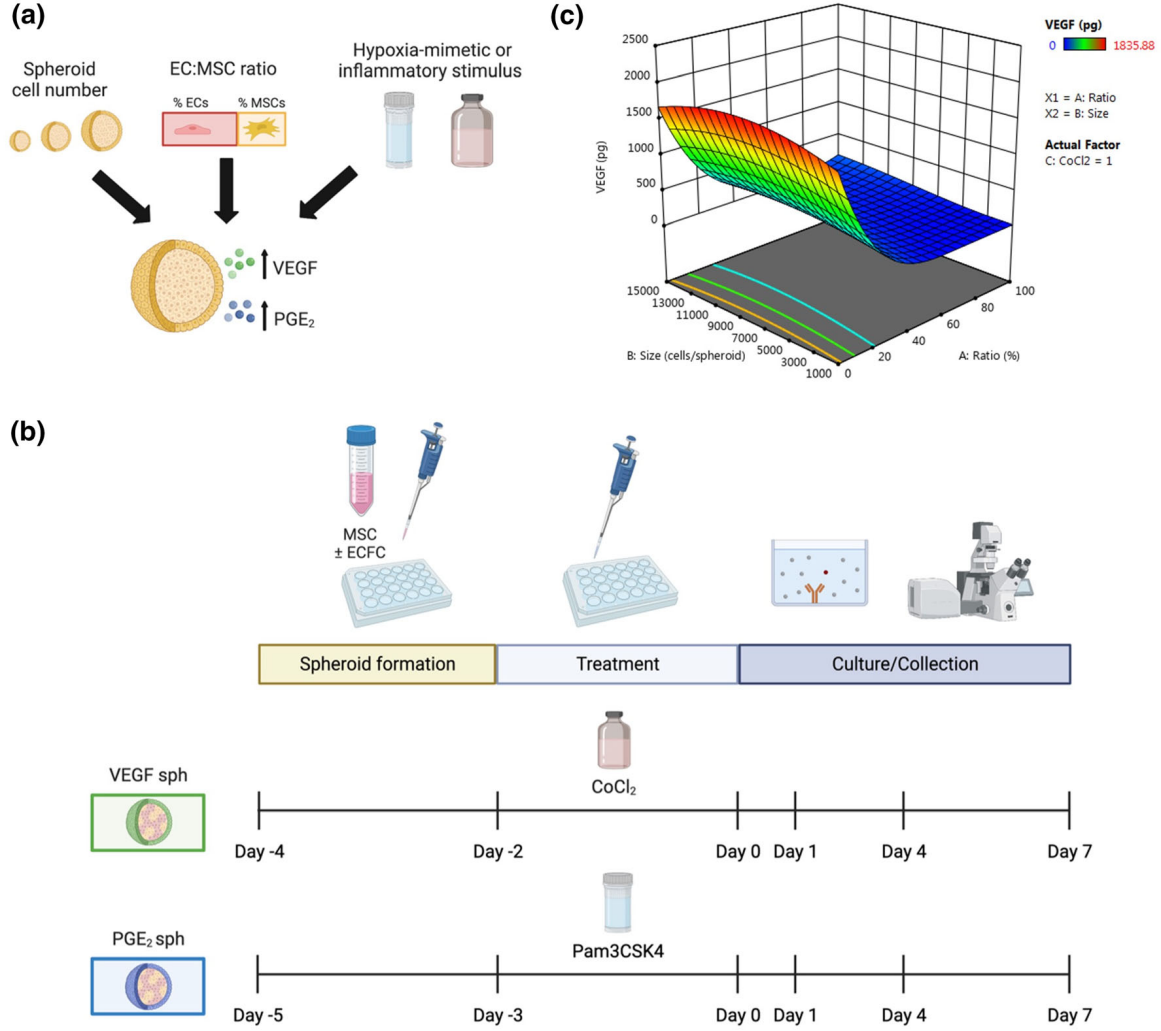
Design and fabrication of distinct MSC spheroids using a DOE approach. (**a**) Microenvironmental conditions were tuned to develop unique MSC spheroids. (**b**) Experimental outline of spheroid formulation. (**c**) 3D response surface map of microenvironmental condition effects on total VEGF secretion from MSC spheroids using 100 µM CoCl_2_.

We used a Box-Behnken experimental design created with Design-Expert software version 11 (Stat-Ease, Minneapolis, MN) to analyze the contribution of three variables (cell number, percentage of ECFCs, and cobalt(II) chloride (CoCl_2_) concentration) on the secretion of VEGF from spheroids (Fig. 1a). These three input variables were chosen due to their ability to modulate the secretome of MSC spheroids. Cell number ranged from 1,000 to 15,000 cells based on enhanced sprouting and sprout length and restored vasculogenic potential of EC-MSC spheroids.^31,34^ The percentage of ECFCs was varied from 0 to 100% where 0% represents spheroids with MSCs alone and 100% represents spheroids with ECs alone. CoCl_2_ (0–100 µM), a hypoxia-mimetic agent, was selected to stabilize HIF-1α and promote the production of pro-angiogenic cytokines.^38^ The significance and interaction of the three input variables on VEGF secretion was assessed with response surface plots generated by the Design-Expert software. PGE_2_ spheroid formulations were designed based on a DOE outlined in our previous work along with the addition of ECFCs. We modeled the effects of cell number and Pam3-Cys-Ser-Lys4 (Pam3CSK4; InvivoGen, San Diego, CA), a bacterial mimetic Toll-like receptor 2 (TLR2) antagonist, on PGE_2_ production.^25^ VEGF and PGE_2_ secretion were measured using cytokine-specific enzyme-linked immunoassay (ELISA) kits according to the manufacturer’s protocol (R&D Systems, Minneapolis, MN).

### Validation of DOE Model and Characterization of Spheroid Secretome

Spheroids were formed from conditions predicted to maximize and minimize secretion of VEGF or PGE_2_ as detailed in Tables 1 and 2, respectively. Culture media was refreshed 24 h prior to collection, and VEGF and PGE_2_ concentrations in conditioned media (CM) were measured by ELISA. The secretory profile of each distinct spheroid was further characterized with a ProcartaPlex™ human angiogenesis panel 18-plex kit to measure angiopoietin-1, BMP-9, EMMPRIN, follistatin, HB-EGF, LYVE-1, TIE-2, CD31, EGF, FGF-2, G-CSF, HGF, IL-8, leptin, PDGF-BB, syndecan, VEGF-A, and VEGF-D (CN: EPX180-15806-901; ThermoFisher) and assessed on the Luminex® xMAP 200 (ThermoFisher). The net mean fluorescence intensity (MFI) was measured and calculated for the 7 standards and samples, and the data were analyzed using the online ProcartaPlex™ Analysis Application.

**Table 1:** Formulations for optimizing VEGF secretion from ECFC-MSC spheroids.

Group	# Cells/spheroid	% ECFCs	CoCl_2_ concentration (µM)
VEGF_MAX_	8,000	0	95.4
VEGF_MIN_	2,850	66	8.8

**Table 2:** Formulations for optimizing PGE_2_ secretion from ECFC-MSC spheroids.

Group	# Cells/spheroid	% ECFCs	Pam3CSK4 concentration (µg/mL)
PGE_2,MAX_	60,000	33	1
PGE_2,MIN_	15,000	33	0

### Spheroid Formation

Combinations of cell number, percentage of ECFCs, and CoCl_2_ concentration were varied to form spheroids that maximized (VEGF_MAX_ and PGE_2,MAX_) and minimized (VEGF_MIN_ and PGE_2,MIN_) VEGF and PGE_2_ secretion using a DOE approach and our previous work.^25^ VEGF_MAX_, VEGF_MIN_, PGE_2,MAX_, and PGE_2,MIN_ spheroid formulations are described in Tables 1 and 2. Spheroids were formed using a forced aggregation method.^32^ Briefly, respective numbers of ECFCs and MSCs were pipetted into 1.5% agarose molds in well plates, and the plates were centrifuged at 500x*g* for 8 min. Plates were maintained in static culture conditions (37 °C, 5% CO_2_, 21% O_2_) for 48 h for spheroid formation in 3:1 EGM-2:α-MEM (3:1 media). Spheroids were then stimulated with either CoCl_2_ or Pam3CSK4 for 72 or 48 h, respectively (Fig. 1b). Media from spheroids was refreshed (1 mL) 24 h prior to collection as CM.

### Characterization of Spheroid Diameter, Cell Distribution, and Mechanical Properties

Prior to spheroid formation, ECFCs were stained with CellTrace™ Violet and MSCs with CellTrace™ Far Red (both from Invitrogen). After two days of spheroid formation, images of the spheroids were acquired via bright-field microscopy (Nikon Eclipse TE2000U). Images were processed and analyzed to quantify spheroid diameter in ImageJ (NIH, Bethesda, MD). Spheroid diameters were determined from analysis of images captured in the xy-plane at the largest cross-sectional area. Confocal images were also taken of the spheroids to visualize the composition of cells in the spheroids (Leica STELLARIS, Leica Camera AG, Wetzlar, Germany). Mechanical characterization of the spheroids was performed using a MicroTester (CellScale, Waterloo, Canada).^13^

### VEGF Bioactivity Validation *via* Scratch Migration and Transwell Migration Assays

We determined the bioactivity of VEGF secreted by MSC spheroids via cell migration assays using ECFCs and diabetic HMVECs. ECFCs were seeded in 24-well plates at 5 × 10^4^ cells/well, and diabetic HMVECs were seeded in 48-well plates at 1.5 × 10^4^ cells/well. Cells were allowed to reach confluency and treated with mitomycin C (5 µg/mL), a cell proliferation inhibitor agent, for 30 min. Mitomycin C was replaced with fresh culture medium, and the confluent monolayer was uniformly scraped with a 200 µL pipette tip. Following wounding, wells were washed with culture medium to remove cell debris, and media was replaced with the respective treatment conditions. Fully supplemented media and 5 ng/mL recombinant VEGF (rVEGF) were used as positive controls while non-supplemented EGM-2 (EGM-2_non-supp_) or EGM-MV2 (EGM-MV2_non-supp_) were used as negative controls for ECFCs or diabetic HMVECs, respectively. Experimental groups consisted of 3:1 1% FBS-containing EGM-2_non-supp_ or EGM-MV2_non-supp_:treatment where treatment was 100:0, 50:50 (or 1:1), or 0:100 VEGF_MAX_:PGE_2,MAX_ CM. A minimum of three regions per scratch were imaged at 10 × magnification with brightfield microscopy immediately and 7 h (for ECFCs) or 6 h 20 min (for diabetic HMVECs) after scratching. Total scratch area was analyzed using ImageJ (NIH), and % EC migration was calculated as follows:

$$\% \;\mathrm{EC\;migration}= \frac{{\mathrm{Total\;scratch\;area}}_{t=0}-{\mathrm{Total\;scratch\;area}}_{t=h}}{{\mathrm{Total\;scratch\;area}}_{t=0}}*100$$

We further evaluated the influence of conditioned media from the distinct spheroids on the migratory activity of endothelial cells in 3D using a transwell migration assay. Transwell cell culture 8 µm FluoroBlok™ inserts (Corning, Corning, NY) were coated with 0.1% gelatin. ECFCs were treated with mitomycin C (5 µg/mL) for 30 min. Mitomycin C was replaced with fresh EGM-2_non-supp_. ECFCs were seeded at 1.28 × 10^5^ cells/well in the upper chamber of the culture insert and allowed to settle for 2 h. Media was added to the lower chamber, where fully supplemented EGM-2 was used as a positive control, EGM-2_non-supp_ was the negative control, and experimental groups consisted of 1:1 EGM-2_non-supp_:CM where CM was 100:0, 50:50 (or 1:1), or 0:100 VEGF_MAX_:PGE_2,MAX_ CM. Cells were incubated for 18 h and then stained with calcein AM (2 µM; ThermoFisher) for 30 min. Migration was determined by capturing fluorescence for each well using a Synergy HTX Multimode Reader (Biotek, Winooski, VT).

### PGE_2_ Bioactivity Validation via Scratch Wound Assay

We assessed the bioactivity of secreted PGE_2_ by scratch wound assays using HaCaTs to determine epithelialization potential of spheroid CM. HaCaTs were seeded in 48-well plates at 4 × 10^4^ cells/well. Cells were cultured for 3 days and treated with mitomycin C (5 µg/mL) for 30 min. Mitomycin C was replaced with fresh culture medium, and cells were uniformly scraped with a 200 µL pipette tip. Following wounding, wells were washed with culture medium to remove any debris, and media was replaced with the respective treatment conditions. Fully supplemented DMEM and 340 pg/mL recombinant PGE_2_ (rPGE_2_) were used as positive controls and non-supplemented DMEM was used as the negative control. Experimental groups consisted of 3:1 1% FBS supplemented DMEM:treatment where treatment was 100:0, 50:50 (or 1:1), or 0:100 VEGF_MAX_:PGE_2,MAX_ CM. A minimum of three regions per scratch were imaged at 10 × magnification with brightfield microscopy immediately and 23 h after scratching. Total scratch area was analyzed using ImageJ (NIH) and % HaCaT migration was calculated as follows:

$$\%\;\mathrm{HaCaT\;migration}= \frac{{\mathrm{Total\;scratch\;area}}_{t=0}-{\mathrm{Total\;scratch\;area}}_{t=23 h}}{{\mathrm{Total\;scratch\;area}}_{\mathrm{t}=0}}*100$$

### PEG-4MAL Hydrogel Synthesis and Spheroid Encapsulation

Four-arm poly(ethylene glycol) (PEG) macromer with maleimide groups at each terminus (PEG-4MAL) (MW 20,000; Laysan Bio, Arab, AL) was dissolved in 4-(2-hydroxyethyl)-1-piperazineethanesulfonic acid) (HEPES) buffer (20 mM in DPBS, pH 7.4). Adhesive and cross-linking peptides were custom synthesized by GenScript (Piscataway, NJ). Adhesive peptide (GRGDSPC, RGD-C, pH 5.5–6) was dissolved in HEPES to generate functionalized PEG-4MAL precursor. Bis-cysteine cross-linking peptide GCRDGPQG↓IAGQDRCG (GPQ-A; ↓ denotes enzymatic cleavage site, pH 4.5 ± 0.1) was dissolved in HEPES at 1:1 maleimide/cysteine ratio after accounting for maleimide groups reacted with adhesive peptide. RGD-C was polymerized to PEG-4MAL macromer at 37 °C for at least 15 min. Spheroid suspensions were added to the functionalized PEG-4MAL precursor to achieve a final concentration of 5 × 10^6^ cells/mL. Gels were individually synthesized by mixing the PEG-4MAL solution with GPQ-A in 8 mm diameter circular silicone molds.^8^ The hydrogels were crosslinked at 37 °C for 20 min to produce 8.0% 20-kDa PEG-4MAL (wt/vol) hydrogels with a final adhesive ligand concentration of 2 mM. Following gelation, the gels were transferred into individual wells of 24-well plates, and the medium was refreshed every 2 days.

### Spheroid Response to Hydrogel Formulations

We assessed cell viability by a Live/Dead assay (ThermoFisher). Metabolic activity was determined using an alamarBlue assay (Invitrogen), with fluorescence read at 590 nm. After 1, 4, and 7 days in culture, spheroids were imaged with brightfield microscopy to assess spheroid spreading in the xy-plane of the scaffold. Spreading of cells from the spheroids was evaluated in ImageJ (NIH) by quantifying the number of protrusions from the spheroids and the protrusion lengths by measuring from the center of the spheroid to the leading edge. These data were calculated on a per spheroid basis where a minimum of 3 spheroids per scaffold were measured and a minimum of 3 scaffolds per group were analyzed. DNA content was measured with the PicoGreen dsDNA assay (Invitrogen).

### Statistical Analysis

Data are presented as means ± standard deviation. Data are derived from a minimum of three independent experiments, and the number of experiments is denoted in the figure legends. Statistical significance was assessed by one-way ANOVA with Tukey’s multiple comparisons test or Student’s t-test when appropriate. *p*-values < 0.05 were considered statistically significant. Statistical analysis was performed using GraphPad Prism® 9 software (GraphPad Software, San Diego, CA). Significance is denoted by alphabetical letterings. Different letters denote statistical significance between groups, while data sharing a letter are not statistically different from one another.

## Results

### DOE Reveals Interplay Between Microenvironmental Conditions and MSC Spheroid Response

We used a DOE approach to determine the significance and interaction of spheroid cell number, percentage of ECFCs, and CoCl_2_ concentration on the secretion of VEGF by MSC spheroids. The secretory potential of the spheroids was dependent on the cell number, percentage of ECs, and CoCl_2_ concentration (Fig. 1c). Spheroid cell number had a weak quadratic relationship with VEGF secretion while percentage of EC and CoCl_2_ concentration demonstrated a positive linear relationship with VEGF secretion. The 3D response surface map generated from the model predicted maximum VEGF secretion (VEGF_MAX_) from spheroids with 8000 cells, 0% ECs, and treated with 95.4 µM CoCl_2_, while minimum VEGF secretion (VEGF_MIN_) from spheroids with 2,850 cells, 66% ECs, and treated with 8.8 µM CoCl_2_ (Table 1).

Spheroids predicted to distinctly maximize and minimize PGE_2_ production were achieved by combining our previously reported work on the optimization of MSC spheroids to enhance anti-inflammatory and proangiogenic potentials and the utilization of co-culture EC-MSC spheroids to enhance capillary network formation.^25^ The number of cells per spheroid and Pam3CSK4 concentration markedly influenced PGE_2_ secretion. Spheroids predicted to maximize PGE_2_ secretion (PGE_2,MAX_) were composed of 60,000 cells and 33% ECs with 1 µg/mL Pam3CSK4, while spheroids predicted to minimize PGE_2_ secretion (PGE_2,MIN_) were comprised of 15,000 cells and 33% ECs with 0 µg/mL Pam3CSK4 (Table 2).

The formulations predicted by the DOE models exemplify the tunability of MSC spheroids and demonstrate the interplay of spheroid cell number, percentage of ECs, and CoCl_2_ or Pam3CSK4 concentration on VEGF or PGE_2_ secretion, respectively. By modulating the microenvironmental conditions in a precise manner, we can effectively tune the secretion of pro-regenerative cytokines from MSC spheroids.

### Cytokine Secretion by MSC Spheroids is Driven by Microenvironmental Conditions

**Figure 2:**
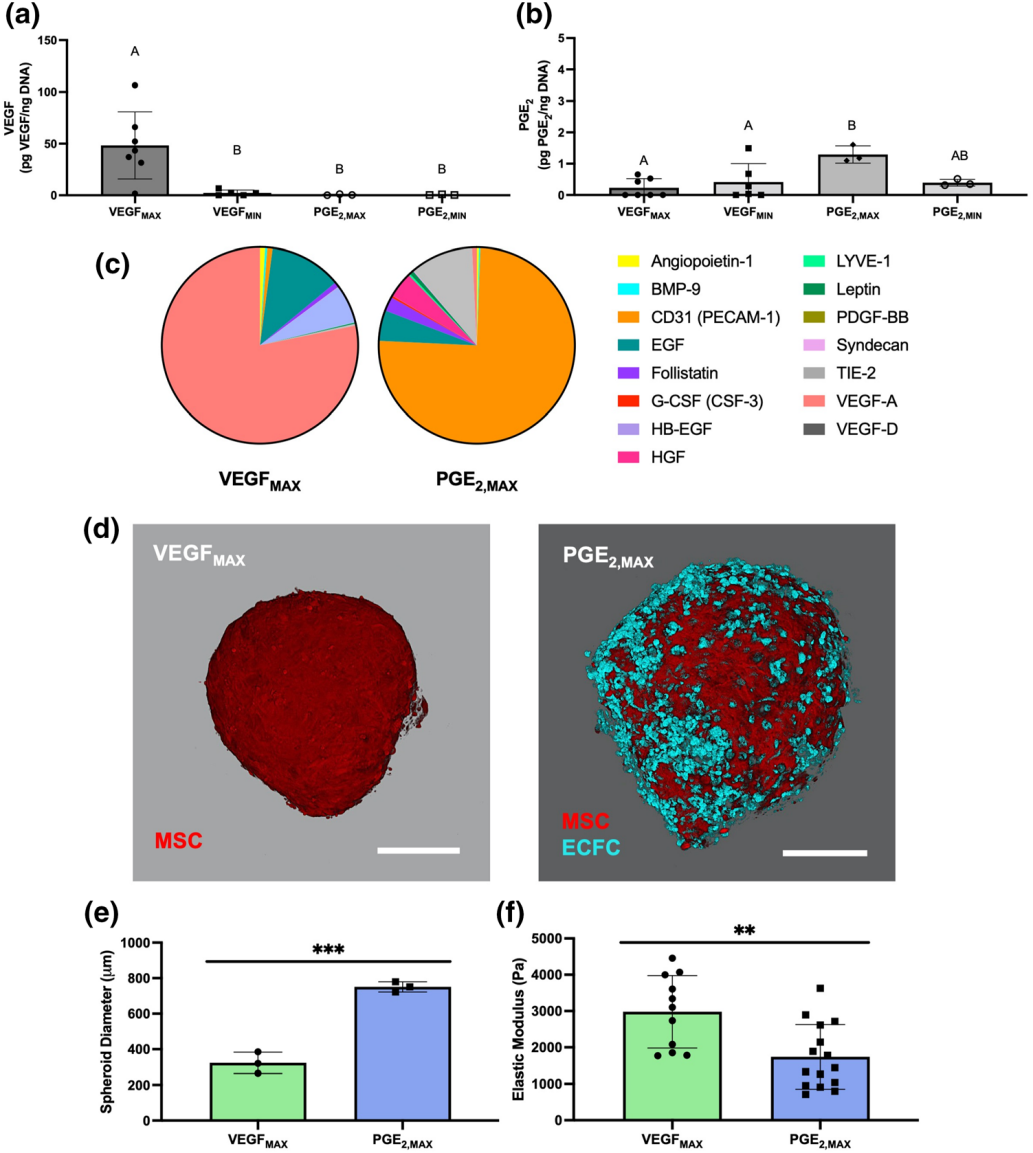
Validation of the DOE model examining cytokine production by the distinct spheroids, and morphological and mechanical characterization of VEGF_MAX_ and PGE_2,MAX_ spheroids. (**a**) Total VEGF secretion and (**b**) total PGE_2_ secretion normalized to total DNA content from spheroid formulations predicted to maximize VEGF (VEGF_MAX_) or PGE_2_ (PGE_2,MAX_) and minimize VEGF (VEGF_MIN_) or PGE_2_ (PGE_2,MIN_) (*n* = 3–6). (**c**) Cytokine content in the secretome of VEGF_MAX_ and PGE_2,MAX_ spheroids. (**d**) Representative VEGF_MAX_ and PGE_2,MAX_ spheroids with ECs (cyan) and MSCs (red) at 48 h post-spheroid formation as observed by confocal microscopy. Scale bars are 200 µm. (**e**) Spheroid diameter (*n* = 3) and (**f**) elastic modulus (*n* = 11–15) of VEGF_MAX_ and PGE_2,MAX_ spheroids at 48 h post-spheroid formation. Significance is denoted by alphabetical letterings or asterisks where different letters (*p* < 0.05) or ** (*p* < 0.01) and *** (*p* < 0.001).

We measured the secretion of VEGF and PGE_2_ to validate DOE formulations predicted to maximize and minimize VEGF and PGE_2_ production. VEGF_MAX_ secreted 21.2-fold more normalized VEGF (48 ± 32 pg VEGF/ng DNA) than VEGF_MIN_ (Fig. 2a). We did not observe significant differences in VEGF secretion among VEGF_MIN_, PGE_2,MAX_, and PGE_2,MIN_ spheroids. Furthermore, PGE_2,MAX_ secreted 3.3-fold more PGE_2_ (1.3 ± 0.3 pg PGE_2_/ng DNA) compared to PGE_2,MIN_ spheroids (Fig. 2b). As expected, PGE_2,MAX_ had significantly elevated PGE_2_ secretion compared to the VEGF counterparts. There were no statistical differences in PGE_2_ secretion among PGE_2,MIN_, VEGF_MAX_, and VEGF_MIN_ spheroids. Collectively, VEGF_MAX_ spheroids had a 90.3-fold increase in normalized VEGF production compared to PGE_2,MAX_ spheroids, and PGE_2,MAX_ spheroids had a 5.6-fold increase in normalized PGE_2_ production compared to VEGF_MAX_ spheroids, validating the formulations extrapolated from the DOEs for these two unique spheroids.

To confirm the reproducibility of these optimized spheroids, we made spheroids from MSCs derived from different donors and measured cytokine secretion. Spheroids made with MSCs from donor 2 (human MSCs from Texas A&M) achieved similar secretion levels as spheroids made with MSCs from donor 1 (human MSCs from RoosterBio) (Supplementary Fig. S1). These data emphasize that the interplay of spheroid cell number, percentage of ECs, and treatment are donor independent and confirm the reproducibility of the formulations identified by the DOE.

We further characterized the secretome profiles of VEGF_MAX_ and PGE_2,MAX_ spheroids (Fig. 2c). Of the analytes examined, the VEGF_MAX_ secretome was primarily composed of VEGF-A, with EGF and HGF and trace amounts of the other analytes. On the other hand, the PGE_2,MAX_ secretome was composed predominantly of CD31, followed by TIE-2, EGF, HGF, and follistatin. VEGF_MAX_ spheroids contained 30-fold more VEGF-A (4098 ± 2490 pg/mL) compared to PGE_2,MAX_ spheroids (136 ± 21 pg/mL). PGE_2,MAX_ spheroids had 293-fold, 129-fold, 2.3-fold, and 1.5-fold more CD31, TIE-2, HGF, and EGF secretion compared to VEGF_MAX_ spheroids, respectively. These data emphasize the different secretory profiles of the distinct spheroid formulations.

### VEGF_MAX_ and PGE_2,MAX_ Spheroids Have Unique Morphologies and Cell Distributions

Following the validation of the predicted formulations, we focused on two spheroids of interest – VEGF_MAX_ and PGE_2,MAX_. After 48 h of spheroid formation, VEGF_MAX_ spheroids appeared slightly elliptical in morphology with tight cell compaction while PGE_2,MAX_ spheroids exhibited a rounder morphology with a close-knit cell arrangement (Fig. 2d). Interestingly, ECs in PGE_2,MAX_ spheroids were unevenly dispersed among the MSCs in the spheroid but were congregated together as multiple cell clusters. PGE_2,MAX_ spheroid diameter was 2.3-fold larger than VEGF_MAX_ spheroids, measuring 750.9 ± 28.6 µm vs. 323.6 ± 59.9 µm (Fig. 2e). The elastic modulus of VEGF_MAX_ spheroids (3674 ± 1924 Pa) was 2.1-fold greater than PGE_2,MAX_ spheroids (1741 ± 887 Pa), a fold increase inversely related to the diameter size (Fig. 2f). Further investigation is merited to explore whether this relationship is due to the difference in spheroid diameter or EC incorporation.

### Functionally Distinct MSC Spheroids Secrete Bioactive Cytokines that Promote EC Migration

**Figure 3:**
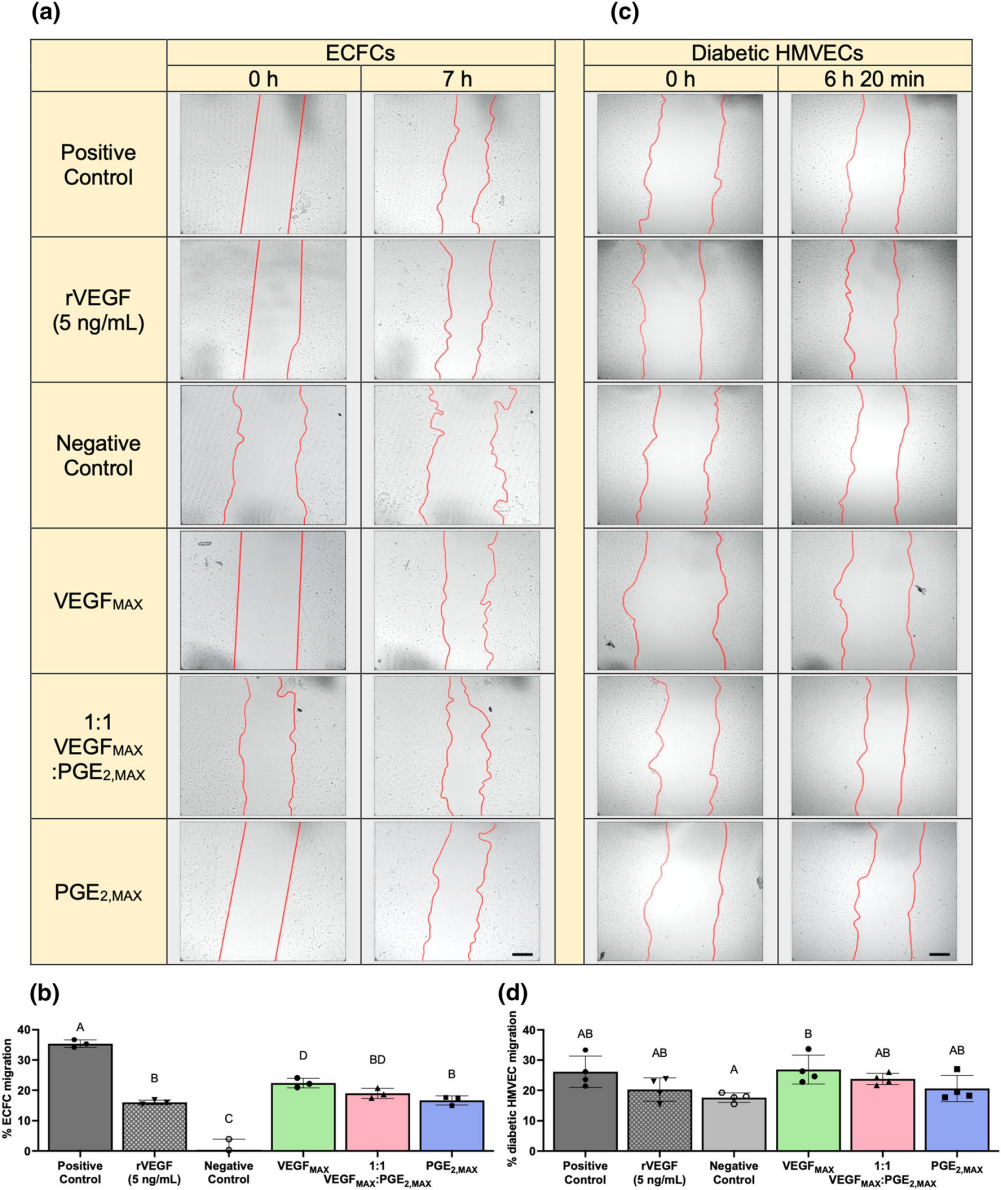
VEGF_MAX_ spheroids secrete bioactive cytokines that enhance EC migration. Images of (**a**) ECFCs and (**c**) diabetic HMVECs in scratch migration assay before and after treatment with CM from VEGF_MAX_, 1:1 VEGF_MAX_:PGE_2,MAX_, PGE_2,MAX_, and 5 ng/mL rVEGF for 7 h or 6 h 20 min, respectively. Fully supplemented and non-supplemented media were used as a positive and negative control, respectively. Red lines indicate the leading edges of the scratch. Scale bars are 200 µm. (**b**) Quantification of % ECFC migration at 7 h (*n* = 3) and (**d**) % diabetic HMVEC migration (*n* = 4) at 6 h 20 min post-scratch and treatment. Different letters denote statistical significance (*p* < 0.05).

We next characterized the functional bioactivity of secreted proangiogenic growth factors by testing the ability of spheroid conditioned media (CM) to stimulate ECFC migration (Fig. 3a). ECFCs were treated with CM from VEGF_MAX_, PGE_2,MAX_, or a combination at a 1:1 ratio. ECFCs in the negative control (0.4 ± 3.5% ECFC migration) exhibited limited migration and receded from the leading edge of the initial scratch, taking on a more rounded morphology (Fig. 3a). In contrast, ECFCs stimulated with CM from VEGF_MAX_ spheroids (22.4 ± 1.6% ECFC migration) demonstrated robust migration, exhibiting 1.3-fold more migration than ECFCs stimulated with CM from PGE_2,MAX_ spheroids (16.6 ± 1.5% ECFC migration) (Fig. 3b). CM composed of a combination of 1:1 VEGF_MAX_ to PGE_2,MAX_ CM (19.0 ± 1.7% ECFC migration) promoted migration at intermediate levels, suggesting the combined CM acts on the ECFCs in an additive manner. VEGF_MAX_ CM treated groups demonstrated less migration compared to the EGM-2 treated (35.4 ± 1.3% ECFC migration) but exhibited 1.4-fold more migration compared to groups treated with rVEGF (16.0 ± 0.8% ECFC migration). These data confirm the functional bioactivity of secreted proangiogenic growth factors from VEGF_MAX_ spheroids. In addition to VEGF, the secretome of VEGF_MAX_ spheroids contains an array of cytokines that act in concert to promote the robust migration of ECFCs.

We also evaluated the migratory response of ECFCs to spheroid CM using a transwell migration assay (Supplementary Fig. S2). CM from VEGF_MAX_ (1039 ± 206 RFU) and PGE_2,MAX_ (1003 ± 239 RFU) induced nearly 5-fold more ECFC migration compared to the negative control, with cell migration similar to the positive control. Unlike in the scratch migration assay, we did not observe significant differences in ECFC migration among groups treated with VEGF_MAX_ and PGE_2,MAX_ CM. This behavior could be attributed to the nature of this assay. Transwell migration assays require low concentrations of chemoattractants to induce migration through the permeable filter. VEGF is a key stimulator of endothelial cell migration during angiogenesis along with other cytokines such as HGF and EGF. VEGF_MAX_ CM contained high levels of VEGF with some HGF and EGF, while PGE_2,MAX_ CM also contained HGF and EGF (Fig. 2c). These data suggest the presence of HGF and EGF in PGE_2,MAX_ CM was sufficient to promote ECFC migration in the transwell.

In chronic wounds, ECs are dysfunctional and unresponsive to potent bioactive factors. To investigate the potency of the spheroid secretomes on rescuing the responsiveness and functionality of ECs from chronic wounds, we tested the effects of our spheroid CM on the migration of diabetic HMVECs (Fig. 3c). Diabetic HMVECs treated with VEGF_MAX_ CM (26.8 ± 4.8% diabetic HMVEC migration) migrated 1.3-fold more than diabetic HMVECs treated with PGE_2,MAX_ CM (20.6 ± 4.3% diabetic HMVEC migration) (Fig. 3d). Combined VEGF_MAX_ and PGE_2,MAX_ CM (23.8 ± 1.9% diabetic HMVEC migration) displayed an additive effect on migration. While not significant, VEGF_MAX_ CM induced more migration than EGM-MV2 (26.1 ± 5.2% diabetic HMVEC migration). Similar to ECFCs, diabetic HMVECs migrated more when treated with VEGF_MAX_ CM compared to rVEGF (20.3 ± 3.9% diabetic HMVEC migration). Collectively, diabetic HMVECs did not exhibit notable differences in migration among groups treated with various CM ratios and rVEGF. However, they did demonstrate similar, although more muted, behavioral trends as the ECFCs, which was anticipated given the impaired functionality of diabetic HMVECs. These findings indicate that VEGF_MAX_ spheroids can partially rescue the functionality of diabetic HMVECs.

### Functionally Distinct MSC Spheroids Secrete Bioactive Cytokines That Promote Epithelialization

**Figure 4:**
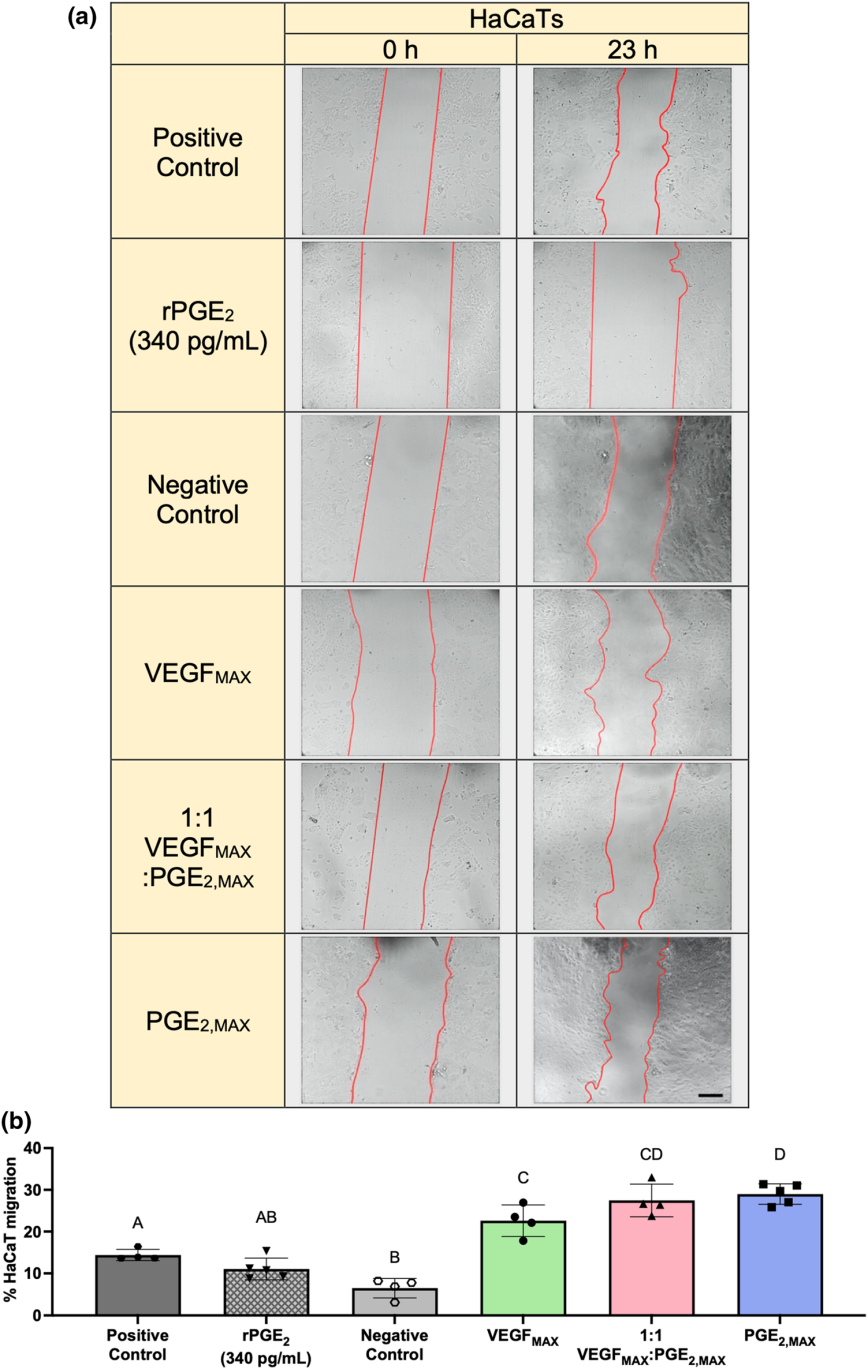
PGE_2,MAX_ spheroids secrete bioactive cytokines that promote keratinocyte migration. (**a**) Images of HaCaTs in scratch wound assay before and after treatment with CM from VEGF_MAX_, 1:1 VEGF_MAX_:PGE_2,MAX_, PGE_2,MAX_, and 340 pg/mL rPGE_2_ for 23 h. Red lines indicate the leading edges of the scratch. Scale bars are 200 µm. (**b**) Quantification of % HaCaT migration at 23 h post-scratch and treatment (*n* = 4). Different letters denote statistical significance (*p* < 0.05).

To characterize the bioactivity of secreted cytokines to promote epithelialization, we tested the ability of spheroid CM to induce HaCaT cell migration (Fig. 4a). HaCaTs were treated with CM from VEGF_MAX_, PGE_2,MAX_, or a combination at a 1:1 ratio. CM from PGE_2,MAX_ spheroids (29.0 ± 2.5% HaCaT migration) induced 1.3-fold more HaCaT migration compared to CM from VEGF_MAX_ spheroids (22.6 ± 3.8% HaCaT migration) (Fig. 4b). The addition of CM from PGE_2,MAX_ spheroids to the CM from VEGF_MAX_ spheroids in the 1:1 VEGF_MAX_:PGE_2,MAX_ (27.5 ± 3.9% HaCaT migration) rescued the migration of HaCaTs. PGE_2,MAX_ CM promoted significantly more HaCaT migration compared to the fully supplemented DMEM positive control while the negative control (6.5 ± 2.3% HaCaT migration) induced limited migration. Intriguingly, HaCaTs treated with rPGE_2_ (11.06 ± 2.60% HaCaT migration) exhibited 2.6-fold less migration compared to PGE_2,MAX_ CM, suggesting that there are other important components in PGE_2,MAX_ CM that jointly promote epithelialization.

### Paracrine Signaling Induces Cellular Protrusions from Spheroids in MMP-Degradable PEG-4MAL Hydrogels

**Figure 5:**
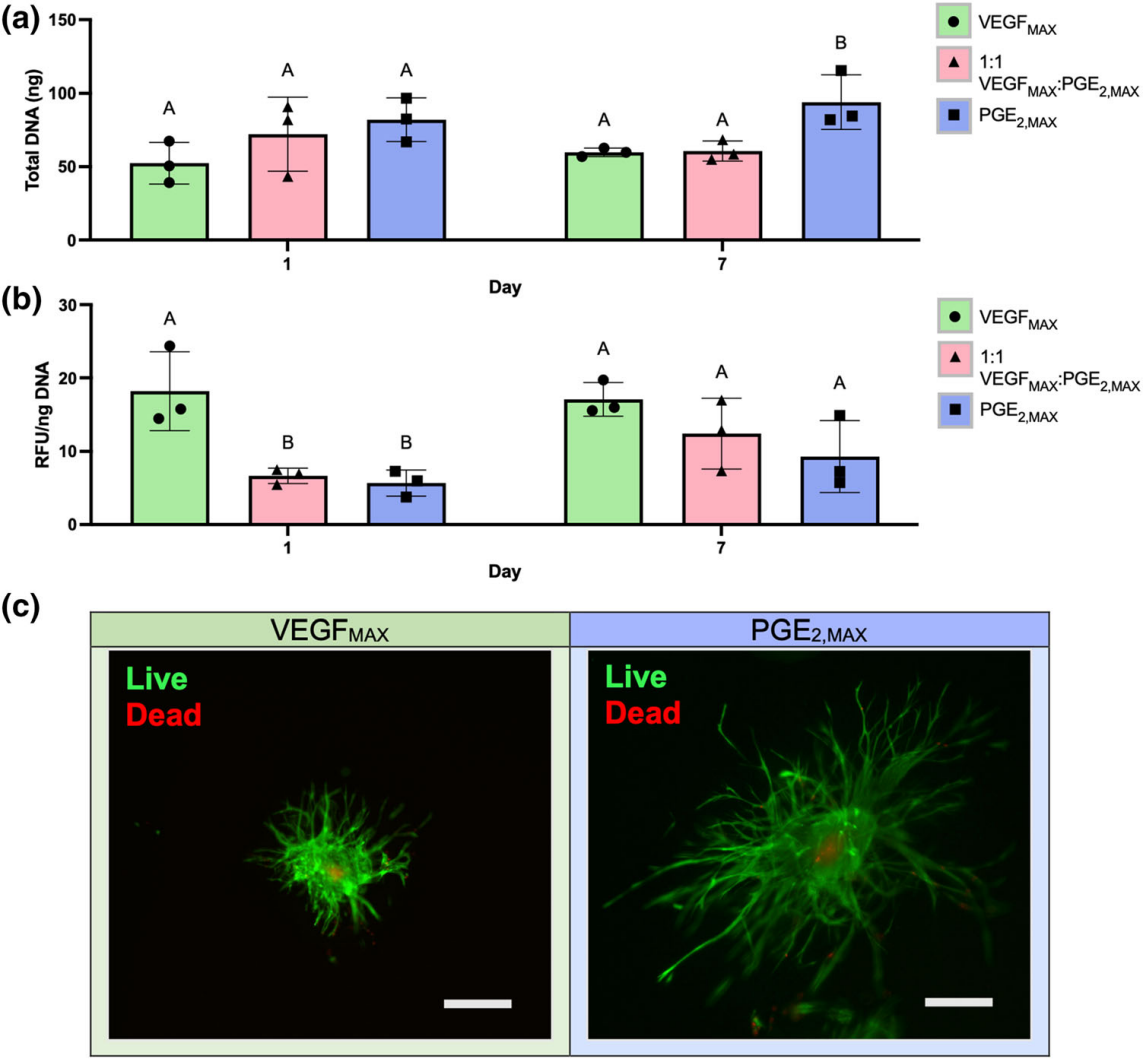
Interaction of functionally distinct spheroids in MMP-sensitive PEG-4MAL hydrogels. (**a**) Total DNA content and (**b**) metabolic activity of spheroids in PEG-4MAL gels on days 1 and 7 (*n* = 3). Statistical analysis was conducted between groups within each time point. Different letters denote statistical significance (*p* < 0.05). (**c**) Live (*green*)/dead (*red*) assay showing the viability of VEGF_MAX_ and PGE_2,MAX_ in 8% PEG-4MAL at day 7. Spheroids were seeded at a ratio of 1:1 VEGF_MAX_:PGE_2,MAX_ spheroids. Scale bar represents 200 µm.

VEGF_MAX_ and PGE_2,MAX_ spheroids were encapsulated in PEG-4MAL hydrogels to study their behavior in a 3D environment. Gels contained either VEGF_MAX_ spheroids, PGE_2,MAX_ spheroids, or 1:1 ratio of VEGF_MAX_:PGE_2,MAX_ spheroids. DNA content remained relatively flat over 7 days for each spheroid ratio (Fig. 5a), suggesting MSCs and ECFCs are not proliferating dramatically over the study duration. On day 1, all groups had similar levels of DNA, ranging between 50 and 85 ng of total DNA. However, on day 7, we detected a 1.6-fold increase in total DNA for gels with only PGE_2,MAX_ spheroids (94.0 ± 18.5 ng DNA) compared to gels with only VEGF_MAX_ (59.8 ± 2.8 ng DNA) or 1:1 VEGF_MAX_:PGE_2,MAX_ (60.7 ± 6.9 ng DNA) spheroids. Similar to DNA content, metabolic activity was consistent over 7 days for each spheroid ratio (Fig. 5b). On day 1, gels with only VEGF_MAX_ spheroids displayed a 2.9-fold increase in metabolic activity compared to PGE_2,MAX_ only (5.7 ± 1.8 RFU/ng DNA) and 1:1 VEGF_MAX_:PGE_2,MAX_ (6.6 ± 1.1 RFU/ng DNA) groups. There were no significant differences among the groups on day 7, although there appeared to be an increase in metabolic activity as the number of VEGF_MAX_ spheroids was increased. These data suggest that PGE_2,MAX_ spheroids proliferated more in PEG-4MAL hydrogels by day 7 compared to other groups, which could be attributed to the MSCs acting on and promoting the proliferation of neighboring ECFCs in the PGE_2,MAX_ spheroid. VEGF_MAX_ only containing gels exhibited a burst in metabolic activity at day 1. This increase may result from an increased energy expenditure by cells to adhere and spread into the gel, as VEGF_MAX_ exhibited a 1.7-fold larger elastic modulus compared to PGE_2,MAX_ spheroids (Fig. 2f).

To investigate cell viability in PEG-4MAL gels, we performed a live/dead stain on spheroids seeded at 1:1 ratio of VEGF_MAX_:PGE_2,MAX_. We confirmed a hypoxic core was not present in PGE_2,MAX_ spheroids due to their large spheroid diameter (Fig. 2e). After 7 days in culture, VEGF_MAX_ and PGE_2,MAX_ entrapped spheroids exhibited similar viability (Fig. 5c), with both spheroid types demonstrating changes in morphology as cells migrated into the gel. Cells in PGE_2,MAX_ spheroids migrated rapidly into the surrounding matrix, developing long, spindle-like protrusions from the spheroid center. The cells were viable with minimal dead cells in the spheroid. Spreading was also prominent, although not as extensive as the cells in the PGE_2,MAX_ spheroids. In these studies, we chose day 7 to assess cellular sprouting during the onset of the proliferation stage. By day 7 of the wound healing cascade, inflammation in the wound bed is typically resolved and proliferation has begun (*i.e.*, ECs and keratinocytes are recruited for angiogenesis and epithelialization, respectively). Future work to study cellular behavior on day 14 would capture spheroid function at later stages in the wound healing cycle.

**Figure 6:**
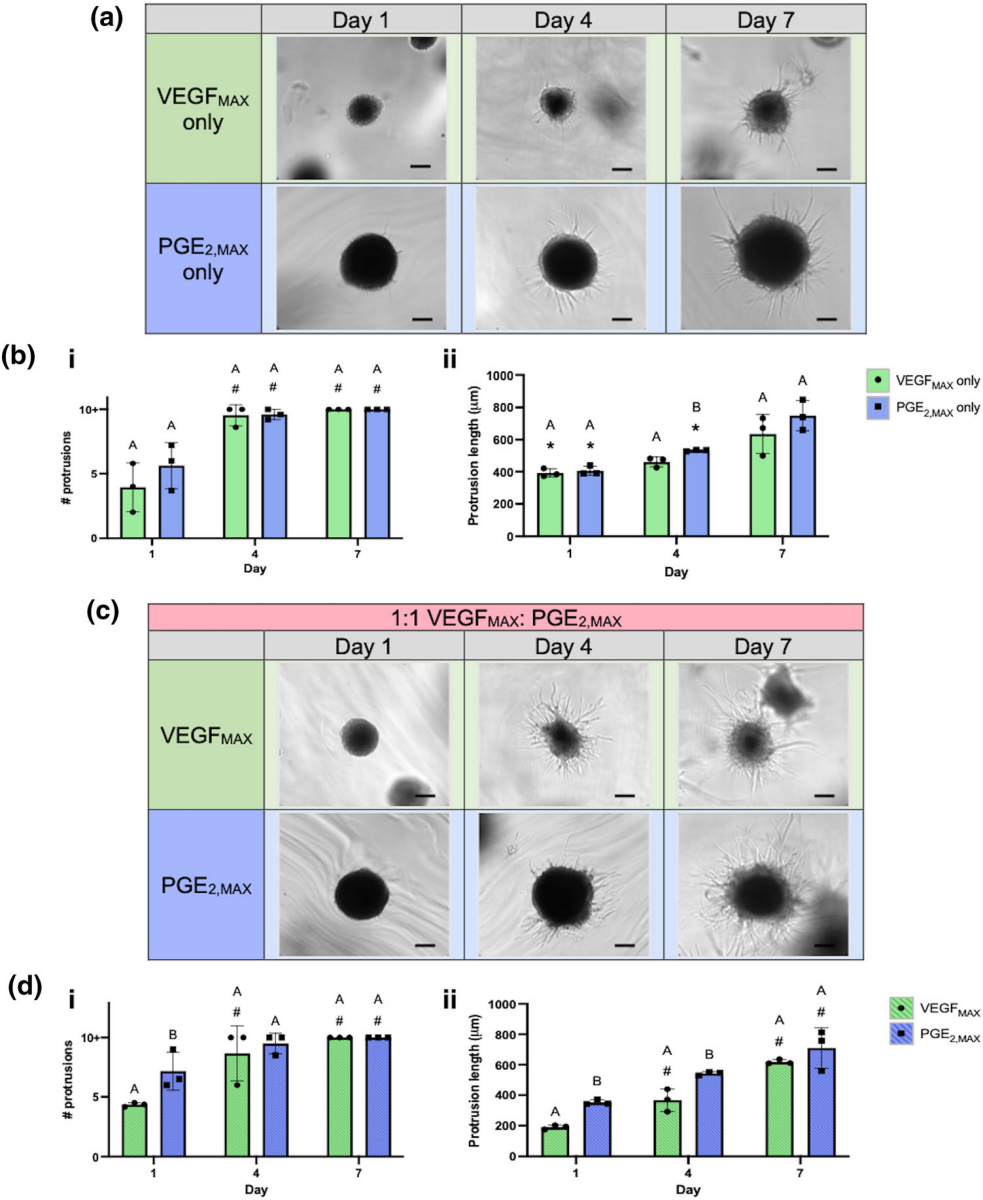
Influence of distinct spheroids on cell spreading in MMP-sensitive PEG-4MAL hydrogels. (**a**) Brightfield images of cell spreading and (**b**) number of protrusions (i) and protrusion length (ii) from VEGF_MAX_ and PGE_2,MAX_ spheroids in gels seeded with only VEGF_MAX_ or PGE_2,MAX_ spheroids on days 1, 4, and 7 (*n* = 3). (**c**) Brightfield images of cell spreading and (**d**) number of protrusions (i) and protrusion length (ii) from VEGF_MAX_ and PGE_2,MAX_ spheroids in gels loaded with spheroids at 1:1 VEGF_MAX_:PGE_2,MAX_ on days 1, 4, and 7 (*n* = 3). * indicates significant differences between that group and day 7 of the same group (*p* < 0.05). # indicates significant differences between that group and day 1 of the same group (*p* < 0.05). Different letters denote statistical differences between groups on designated days (*p* < 0.05). Scale bars represent 200 µm.

The MMP-sensitivity of the PEG-4MAL hydrogels facilitates gel degradation by spheroid-secreted MMPs, providing a scaffold to investigate the interaction of the unique MSC spheroids with their microenvironment. We assessed cell spreading from the spheroids in the gels by quantifying the number of protrusions and the protrusion lengths (Fig. 6b i,ii). On day 1, PGE_2,MAX_ spheroids exhibited 1.4-fold more protrusions than VEGF_MAX_ spheroids, with no differences in protrusion lengths (390–410 µm) (Fig. 6a). However, on day 4, we observed the opposite trend with no apparent distinctions in the number of protrusions but significant differences in protrusion length-PGE_2,MAX_ spheroids (532 ± 5 µm) had 1.2-fold longer protrusions compared to VEGF_MAX_ spheroids (461 ± 31 µm). This behavior was maintained on day 7 when the number of protrusions for VEGF_MAX_ only and PGE_2,MAX_ only hydrogel groups exceeded 10 and PGE_2,MAX_ (750 ± 94 µm) had 1.2-fold longer protrusions compared to VEGF_MAX_ (635 ± 121 µm). For both groups, the number of protrusions and protrusion length increased over 7 days. These data demonstrate that PEG-4MAL scaffolds enable cellular protrusions from VEGF_MAX_ and PGE_2,MAX_ spheroids, evidenced by the increasing number of protrusions and protrusion length over time.

To examine the influence of spheroid type on cell spreading, we quantified protrusion number and protrusion length from VEGF_MAX_ and PGE_2,MAX_ spheroids when seeded at 1:1 ratio of VEGF_MAX_:PGE_2,MAX_ spheroids in PEG-4MAL hydrogels (Fig. 6d i,ii). Over 7 days, the number of protrusions and protrusion length increased for both VEGF_MAX_ and PGE_2,MAX_ spheroids (Fig. 6c). PGE_2,MAX_ spheroids (353 ± 16 µm, day 1; 543 ± 12 µm, day 4) had significantly longer protrusions than VEGF_MAX_ spheroids (191 ± 13 µm, day 1; 368 ± 74 µm, day 4), which plateaued by day 7. Interestingly, the combination of the two distinct spheroids increased the number of protrusions on day 1 in VEGF_MAX_ spheroids by 1.1-fold and PGE_2,MAX_ spheroids by 1.3-fold more compared to the respective spheroids in the VEGF_MAX_ only or PGE_2,MAX_ only gels while the protrusion length remained unaffected. These differences were less apparent by day 4 and 7 for both spheroid types. Together, these data suggest that increases in protrusion number for each unique spheroid on day 1 is related to paracrine signaling between the spheroids.

## Discussion

MSCs are widely studied to promote wound healing and tissue regeneration due to their abundant therapeutic potential and their potent secretomes.^1,19^ We and others previously reported the development of MSC spheroids that enhance VEGF and PGE_2_ production compared to MSCs cultured in monolayer.^21,25,37^ However, this approach is limited by the dependence on host ECs for neovascularization, posing a challenge as ECs in chronic, nonhealing wounds are unresponsive to potent bioactive factors in their environment.^10^ Furthermore, the simultaneous induction of VEGF and PGE_2_ secretion from a single spheroid inhibits the ability to fully capitalize on the therapeutic capabilities of MSCs. In this study, we addressed these challenges by engineering MSC spheroids with distinct therapeutic potentials (*i.e.*, maximizing VEGF or PGE_2_ secretion) and incorporating ECFCs to promote vascularization and epithelialization for wound repair without dependence on native ECs.

This study established that the distinct MSC spheroids possess unique bioactive functionalities. Using DOE, three microenvironmental conditions were tuned to engineer MSC spheroids with various VEGF (VEGF_MAX_ and VEGF_MIN_) or PGE_2_ (PGE_2,MAX_ and PGE_2,MIN_) secretion levels. The input variables (*i.e.*, number of cells per spheroid, percentage of ECFCs, and CoCl_2_ or Pam3CSK4 concentration) were chosen due to their ability to modulate the secretome of MSC spheroids.^25,31,34,35^ Spheroid cell number had a weak quadratic relationship with VEGF secretion while percentage of ECs and CoCl_2_ concentration had a positive linear influence on VEGF levels. On the other hand, PGE_2_ secretion levels increased with spheroid cell number and Pam3CSK4 concentration. Using a multiplex cytokine assay, we further characterized the spheroid secretome and established the unique profile compositions of the optimized formulations. Interestingly, CM from either VEGF_MAX_ or PGE_2,MAX_ spheroids promoted more migration than the respective recombinant cytokines when applied to ECFCs, diabetic HMVECs, or HaCaTs in a scratch migration/wound assay. Taken together, these data confirm that the CM from VEGF_MAX_ and PGE_2,MAX_ spheroids is comprised of not only VEGF or PGE_2_, respectively, but rather a cocktail of potent factors that work synergistically to enhance angiogenesis and epithelialization. These data also highlight the capability of leveraging the spheroids in a modular fashion.

The cell density of the PGE_2,MAX_ spheroids (60 k cells/spheroid) represents a potential limitation with this approach. When applied clinically, the number of spheroids would need to be scaled up, which could be challenging given the number of MSCs required. We also recognize that other environmental factors (*e.g.*, physiological oxygen tension in the skin) influence the behavior of MSC spheroids, which merits further investigation. Non-steroidal anti-inflammatory drugs (NSAIDs) block cyclooxygenase, which inhibit PGE_2_ production. Further exploration is necessary to better understand the effects of NSAIDs on the therapeutic potential of PGE_2,MAX_ spheroids, as the clinical translation of this approach may coincide with NSAID use. Nonetheless, we demonstrated that we can selectively upregulate the production of specific cytokines in different MSC spheroids using a DOE approach. Furthermore, by independently tuning MSC spheroids, we facilitated paracrine interaction and spheroid crosstalk for therapeutic applications.

MMPs play a critical role in regulating extracellular matrix (ECM) degradation and deposition that is required for epithelialization during cutaneous wound healing.^27^ However, chronic wounds are often characterized by an excess amount of MMPs that dysregulate the balance between granulation tissue formation and re-epithelialization.^4^ Furthermore, MSC spheroids exhibit increased MMP secretion that facilitates cell spreading and migration.^7^ We investigated the interaction of the distinct spheroids using an MMP-degradable PEG-based platform based on its high cytocompatibility and minimal inflammation* in vivo*. PEG-4MAL gels are well characterized and facilitate robust cellular engraftment and growth.^11,26^ In addition, the synthetic nature of PEG-4MAL enables high mechanical tunability and the incorporation of elements characteristic of natural ECM, such as cell-adhesion motifs and sites vulnerable to proteases to regulate matrix remodeling during wound healing, which are more difficult to control with natural biomaterials. Overall, the ease of manipulating PEG-4MAL hydrogels is advantageous when considering this platform for clinical translation. PEG-4MAL scaffold degradation can be regulated by tuning the concentration of protease susceptible peptides to facilitate matrix remodeling and accelerate wound healing. VEGF_MAX_ and PGE_2,MAX_ spheroids in PEG-4MAL scaffolds maintained viability over 7 days. There was a burst in metabolic activity at day 1 for hydrogels loaded with only VEGF_MAX_ spheroids and more pronounced proliferative activity for groups containing PGE_2,MAX_ spheroids (PGE_2,MAX_ and 1:1 VEGF_MAX_:PGE_2,MAX_ groups). The burst in metabolic activity at day 1 for gels loaded with only VEGF_MAX_ spheroids may be due to the size and elastic modulus of the spheroids. VEGF_MAX_ spheroids are 43% smaller in diameter and possess a 2.1-fold greater elastic modulus compared to PGE_2,MAX_ spheroids. The more compact size and greater cell–cell adhesion in the VEGF_MAX_ spheroid would require more effort from the MSCs to attach and spread into the surrounding scaffold. This is supported by the smaller number of cell protrusions for VEGF_MAX_ spheroids compared to PGE_2,MAX_ spheroids. MSCs have potent secretomes that act on neighboring cells. Due to their proximity and potential cell interactions between the ECs and MSCs in PGE_2,MAX_ spheroids, the observed increase in DNA content using these spheroids may be attributed to EC proliferation.^3^ In these studies, measurements of cellular protrusions were performed on individual z-planes, which restricted our evaluation to two dimensions. Future work could include capturing the cellular protrusions in the z-direction to assess radial spreading and characterize the direction and alignment of cellular outgrowth.

Current cellular or pharmacological approaches for wound healing are limited because they fail to consider the range of clinical challenges in chronic wounds (*e.g.*, angiogenesis, epithelialization, and regulation of matrix remodeling).^5,22^ Herein, we developed a clinically relevant approach using functionally distinct MSC spheroids and established their effectiveness at promoting angiogenesis and epithelialization. The development of functionally distinct MSC spheroids offers the potential to apply them in a modular fashion. However, when used together, the unique spheroids have a positive interaction as demonstrated by the initial elevated number of cell protrusions. Future studies are warranted to investigate the role of MSC spheroid ratio on wound repair. Furthermore, we loaded the MSC spheroids into a biocompatible, highly tunable PEG-4MAL hydrogel to provide a controlled environment to investigate cell attachment and spreading. We utilized a slow-degrading PEG-4MAL gel formulation because we sought to understand the microenvironmental and spheroid-spheroid interactions of our functionally distinct spheroids. Future work to study the influence of gels with varying degradation rates would advance our knowledge on how matrix remodeling impacts cutaneous wound repair. In addition, the application of VEGF_MAX_ and PGE_2,MAX_ spheroids to an* ex vivo* or* in vivo* wound model would deepen our understanding of the therapeutic benefits of these spheroids, but this is beyond the scope of the present work.

We engineered functionally distinct MSC spheroids to promote angiogenesis or epithelialization by maximizing VEGF or PGE_2_ secretion, respectively. These MSC spheroids represent an innovative strategy to leverage the unique functionality of spheroids in a modular fashion for the treatment of cutaneous wounds or other tissue deficits. This approach, for the first time, incorporates ECs as building blocks to facilitate vascularization without dependence on host ECs. Collectively, VEGF_MAX_ and PGE_2,MAX_ spheroids deployed in an MMP-degradable PEG-4MAL hydrogel hold promise as a safe, effective treatment for wound healing or other clinical challenges that require effective neovascularization.

## Supplementary Information

Below is the link to the electronic supplementary material.

Unique MSC spheroid formulations are independent of MSC donor type (*n* = 3). Donor 1 is human MSCs from RoosterBio and donor 2 is human MSCs from Texas A&M. VEGF or PGE_2_ production by the spheroid formulations predicted to maximize VEGF (VEGF_MAX_) or PGE_2_ (PGE_2,MAX_) and minimize VEGF (VEGF_MIN_) or PGE_2_ (PGE_2,MIN_) were measured via ELISA. (A) Total VEGF secretion, (B) total VEGF secretion normalized to total DNA content, (C) total PGE_2_ secretion, and (D) total PGE_2_ secretion normalized to total DNA content from VEGF_MAX_, VEGF_MIN_, PGE_2,MAX_, PGE_2,MIN_ spheroids formulated with different MSC donors (*n* = 3-6). Significance is denoted by alphabetical letterings; different letters denote statistical significance (*p *< 0.05). Supplementary file1 (TIFF 15267 kb).

VEGF_MAX_ and PGE_2,MAX_ secrete bioactive cytokines that promote the migration of ECFCs in a transwell migration assay (*n* = 3). Significance is denoted by alphabetical letterings; different letters denote statistical significance (*p *< 0.05). Supplementary file2 (TIFF 145 kb).

## Abbreviations

ECFC: Endothelial colony forming cells
MSC: Mesenchymal stromal cells
CoCl_2_: Cobalt(II) chloride
VEGF: Vascular endothelial growth factor
VEGF_MAX_: Spheroids that maximize VEGF production
VEGF_MIN_: Spheroids that minimize VEGF production
PGE_2_: Prostaglandin E2
PGE_2,MAX_: Spheroids that maximize PGE_2_ production
PGE_2,MIN_: Spheroids that minimize PGE_2_ production
EGF: Epidermal growth factor
CD31 (PECAM-1): Cluster of differentiation 31 (platelet endothelial cell adhesion molecule)
HGF: Hepatocyte growth factor
PEG-4MAL: 4-arm poly(ethylene glycol) maleimide
MMP: Matrix metalloproteinase
DOE: Design of experiments
