# Supplementary figures and images for: Tuning the Microenvironment to Create Functionally Distinct Mesenchymal Stromal Cell Spheroids
Source: Ann Biomed Eng. 2023 Feb 21;51(7):1558–73. doi: 10.1007/s10439-023-03162-9 (PMC10264490; doi:10.1007/s10439-023-03162-9)

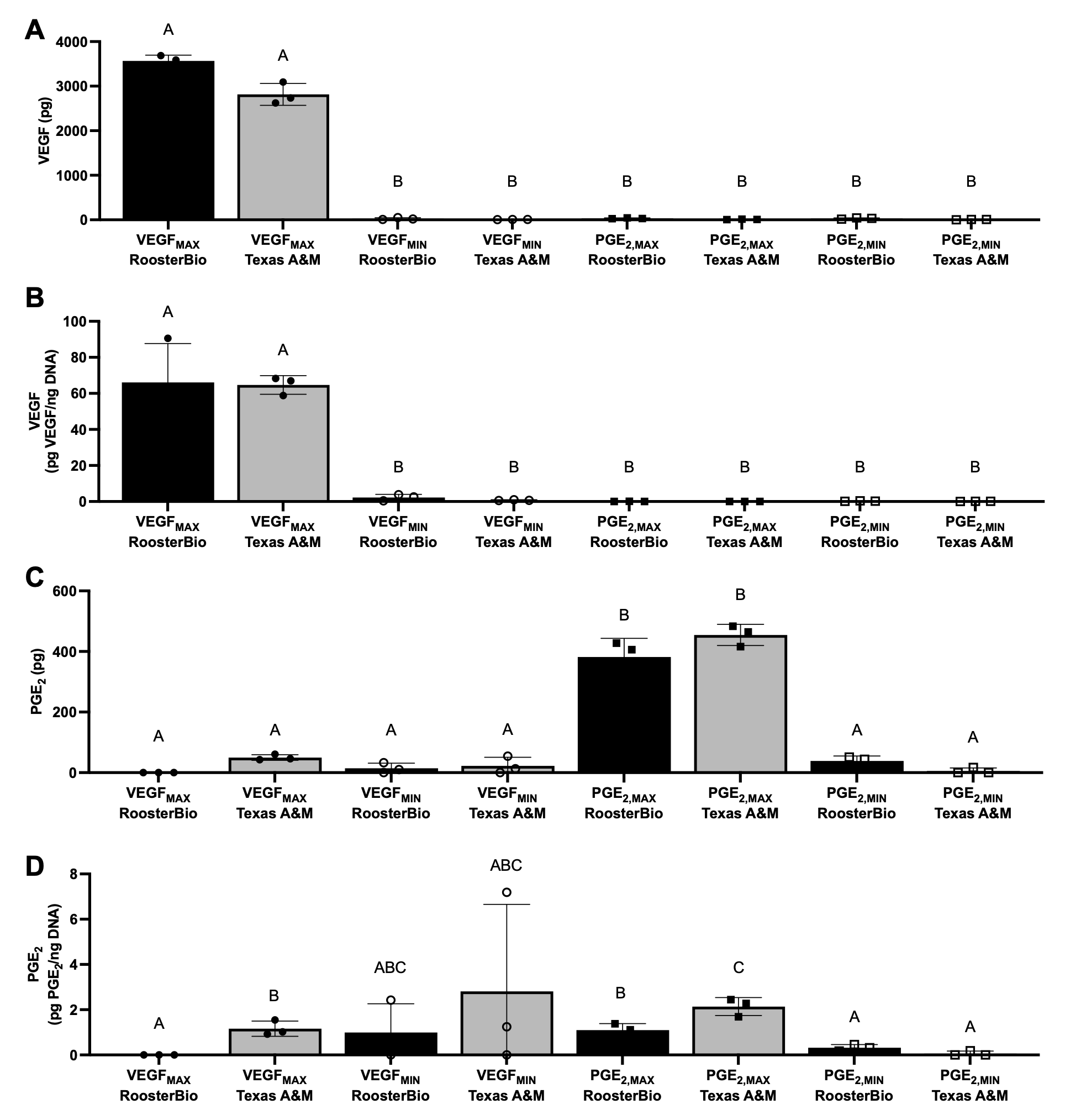

Supplement: Supplementary file 1 — Unique MSC spheroid formulations are independent of MSC donor type (n = 3). Donor 1 is human MSCs from RoosterBio and donor 2 is human MSCs from Texas A&M. VEGF or PGE2 production by the spheroid formulations predicted to maximize VEGF (VEGFMAX) or PGE2 (PGE2,MAX) and minimize VEGF (VEGFMIN) or PGE2 (PGE2,MIN) were measured via ELISA. (A) Total VEGF secretion, (B) total VEGF secretion normalized to total DNA content, (C) total PGE2 secretion, and (D) total PGE2 secretion normalized to total DNA content from VEGFMAX, VEGFMIN, PGE2,MAX, PGE2,MIN spheroids formulated with different MSC donors (n = 3-6). Significance is denoted by alphabetical letterings; different letters denote statistical significance (p < 0.05). Supplementary file1 (TIFF 15267 kb). [file 10439_2023_3162_MOESM1_ESM.tiff]

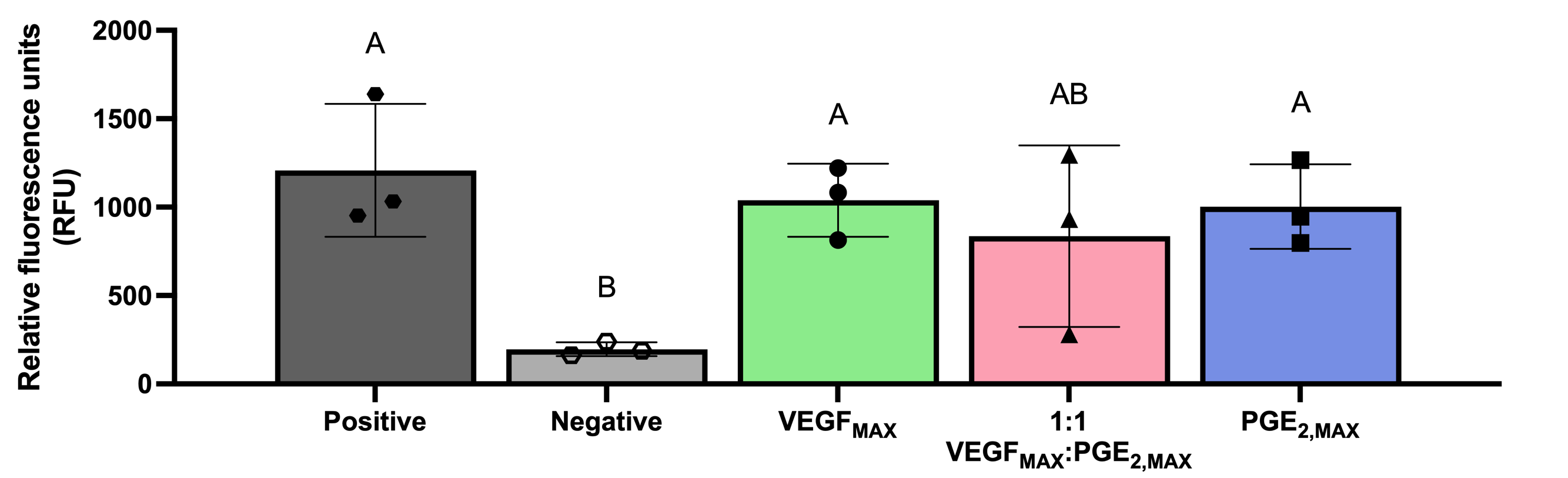

Supplement: Supplementary file 2 — VEGFMAX and PGE2,MAX secrete bioactive cytokines that promote the migration of ECFCs in a transwell migration assay (n = 3). Significance is denoted by alphabetical letterings; different letters denote statistical significance (p < 0.05). Supplementary file2 (TIFF 145 kb). [file 10439_2023_3162_MOESM2_ESM.tiff]
